# Trees Grow on Money: Urban Tree Canopy Cover and Environmental Justice

**DOI:** 10.1371/journal.pone.0122051

**Published:** 2015-04-01

**Authors:** Kirsten Schwarz, Michail Fragkias, Christopher G. Boone, Weiqi Zhou, Melissa McHale, J. Morgan Grove, Jarlath O’Neil-Dunne, Joseph P. McFadden, Geoffrey L. Buckley, Dan Childers, Laura Ogden, Stephanie Pincetl, Diane Pataki, Ali Whitmer, Mary L. Cadenasso

**Affiliations:** 1 Department of Biology, Northern Kentucky University, Highland Heights, Kentucky, United States of America; 2 Department of Economics, College of Business and Economics (COBE), Boise State University, Boise, Idaho, United States of America; 3 School of Sustainability, Arizona State University, Tempe, Arizona, United States of America; 4 Center for Eco-Environmental Sciences, Chinese Academy of Sciences, Haidian District, Beijing, China; 5 Department of Forestry and Environmental Resources, North Carolina State University, Raleigh, North Carolina, United States of America; 6 USDA Forest Service, Northern Research Station, Baltimore, Maryland, United States of America; 7 University of Vermont, Rubenstein School of Environment and Natural Resources and Spatial Analysis Lab, Burlington, Vermont, United States of America; 8 Department of Geography, University of California Santa Barbara, Santa Barbara, California, United States of America; 9 Department of Geography, Ohio University, Clippinger Laboratories 109, Athens, Ohio, United States of America; 10 Department of Global and Sociocultural Studies, Florida International University, FIU Modesto A. Maidique Campus, Miami, Florida, United States of America; 11 Institute of the Environment and Sustainability, University of California Los Angeles, Los Angeles, California, United States of America; 12 Department of Biology, University of Utah, Salt Lake City, Utah, United States of America; 13 Georgetown University, Washington D.C., United States of America; 14 Department of Plant Sciences, University of California Davis, Davis, California, United States of America; University of Siena, ITALY

## Abstract

This study examines the distributional equity of urban tree canopy (UTC) cover for Baltimore, MD, Los Angeles, CA, New York, NY, Philadelphia, PA, Raleigh, NC, Sacramento, CA, and Washington, D.C. using high spatial resolution land cover data and census data. Data are analyzed at the Census Block Group levels using Spearman’s correlation, ordinary least squares regression (OLS), and a spatial autoregressive model (SAR). Across all cities there is a strong positive correlation between UTC cover and median household income. Negative correlations between race and UTC cover exist in bivariate models for some cities, but they are generally not observed using multivariate regressions that include additional variables on income, education, and housing age. SAR models result in higher r-square values compared to the OLS models across *all* cities, suggesting that spatial autocorrelation is an important feature of our data. Similarities among cities can be found based on shared characteristics of climate, race/ethnicity, and size. Our findings suggest that a suite of variables, including income, contribute to the distribution of UTC cover. These findings can help target simultaneous strategies for UTC goals and environmental justice concerns.

## Introduction

The environmental justice (EJ) community, including activists, academics, and policy makers, has long been concerned with recognized patterns of low-income and minority communities disproportionately burdened by environmental hazards [1,2]. Recently, the scope of environmental equity has been broadened from a sole focus on burdens to include the distribution of environmental goods or amenities [3]. To date, studies on the allocation of amenities across urbanizing landscapes have focused mostly on the placement of parks and open spaces [4–7], as well as vegetation cover [8–10]. Generally these studies report uneven distribution of environmental amenities that disfavor racial and ethnic minority and low income neighborhoods.

Environmental justice studies typically focus on single case studies. This reflects the grassroots and local nature of environmental justice struggles that have informed analytical approaches [11]. However, comparative analyses of multiple cities using the same methodology can reveal whether there are consistent and significant patterns across cities of different sizes, morphologies, biophysical contexts, ages of development, and histories. Although comparative ecological methodologies have been promoted in the field of urban ecology, comparative urban environmental justice studies are relatively few [12–15] and tend to focus on measuring environmental burdens rather than benefits [16,17]. Such analyses on generalizable patterns of environmental inequity should ideally lead to investigations of processes responsible for common and differing patterns observed, including the role of historic, place-specific drivers of urbanization and environmental change [11,18]. Before such process-based studies can be undertaken, however, investigators should carefully examine the distributive equity of benefits or burdens.

Urban Tree Canopy (UTC) cover is widely regarded as an environmental good or amenity. UTC cover as an environmental amenity stems from the direct flow of perceived benefits, or ecosystem services, to people and neighborhoods where UTC cover is found. UTC cover has been linked to the provisioning of multiple ecosystem services, including regulation of regional climate and water cycles [19–26]. In addition to UTC, “greenness”, as an indicator of vegetation cover, has been associated with reductions in childhood obesity rates [27]. Presence of vegetation can also decrease cognitive fatigue, improve worker attitudes on the job, and reduce stress as well as feelings of anger, depression, or anxiety [28,29]. UTC cover has been associated with improved aesthetics, noise reduction, and stronger social cohesion and community empowerment [19,30–32]. It is important to note, however, that UTC can also create disamenities such as increased water demand, maintenance costs, allergies, and perceived safety concerns [33,34]. Some street tree campaigns have been met with resistance from residents who simply do not want trees in front of their houses. The potential costs or burdens of UTC cover depend on a number of factors, including climate, vulnerability and price of water supply in a changing climate, socio-demographic preferences, built environment characteristics, and fiscal capacity to maintain UTC cover [35]. While benefits from and support for UTC are not universally positive, for the purposes of this analysis, we treat UTC as an environmental amenity.

Fairness of public investment in the distribution, delivery, and maintenance of services derived from urban tree canopies is a basic environmental justice concern [19,27,30,31,33,36]. Equity assessments must take into account existing distributions of benefits as they relate to social groups, especially ethnic/racial minorities and lower-income groups who have been traditionally disadvantaged and marginalized, or lack the resources or capacity to overcome a scarcity of environmental benefits [37]. These considerations point to the need for UTC assessments at a scale smaller than municipal jurisdictions because distributional patterns likely vary depending on the scale of analysis [38]. Research must match the scale of analysis to the social processes that drives the distribution of amenities in order to examine patterns at scales meaningful to environmental justice inquiries. Important social processes may occur at the scale of a neighborhood, a defined territory that reflects and reinforces group belonging. In general, studies relating UTC cover to demographics and race use census tracts as a proxy for neighborhood. For this paper, we use the census block group (CBG), a subset of the census tract, as the unit of analysis. We tested for scale differences using the census tract and census block group as analytical units but did not find meaningful differences between the two.

The spatial resolution of biogeophysical datasets, similar to census data, is predicted to affect UTC assessments, a phenomenon that is referred in spatial sciences as the Modifiable Areal Unit Problem [39]. Individual trees or lines of street trees—both common in urban areas—are not captured by moderate-resolution imagery [40]. Historically, moderate-resolution data derived from 30 meter resolution Landsat Thematic Mapper imagery has been used to classify tree canopy cover. These moderate resolution datasets, such as the National Land Cover Dataset (NLCD) [41], have allowed for nation-wide comparisons, but their usefulness is limited in urban systems that exhibit fine spatial heterogeneity [42]. The advancement of high resolution imagery and adoption of geographic information systems (GIS) by government entities has resulted in an abundance of high resolution geospatial data that can be used to derive very high (< 1m) spatial resolution vegetation data [43] including individual tree canopies. Accurate land cover data from high resolution imagery may help uncover patterns in UTC cover masked by the NLCD. Still, even with higher spatial resolution, important issues such as tree species appropriateness for different climate zones remains difficult to ascertain [44].

High spatial resolution of social and biogeophysical datasets matter little if the appropriate statistical analyses are not used. Some statistical methodologies used in ecology do not account for the spatial structure of data. For example, ordinary least squares (OLS) regression is a common technique to assess relationships between dependent and predictor variables, yet it fails to account for spatial autocorrelation, which may violate the assumption of independence of the errors in the regression. Failure to account for spatial autocorrelation can result in biased regression estimates due to a lack of accounting of spatial dependence in variables; or it can lead to higher standard errors in the regression estimators affecting statistical inference [45]. Alternatively, spatial autoregression (SAR) models account for spatial autocorrelation and therefore correct for violations of the assumptions in the classic linear regression models. The importance of SAR models has been widely demonstrated in various social and natural science fields and at their intersection [46,47].

In this study we examine potential inequities associated with the distribution of urban tree cover in relationship to race/ethnicity and income, in seven cities across the US: Baltimore, MD, Los Angeles, CA, New York, NY, Philadelphia, PA, Raleigh, NC, Sacramento, CA, and Washington, DC. This work is the product of a NCEAS (National Center for Ecological Analysis and Synthesis) working group on the urban ecology of environmental justice. NCEAS working groups focus on discovering novel patterns in existing datasets rather than creation of new datasets. The NCEAS participants chose these cities based on their expert knowledge and availability of high spatial resolution UTC cover. We hypothesized that urban tree cover would be positively correlated with increasing income and negatively correlated with minority populations in all cities. In particular, we anticipated that bivariate analyses would indicate a statistically significant and strong negative relationship between tree cover and minority populations, but the use of multivariate regressions incorporating control variables such as population density, housing age, education, and income would change that relationship. As all of our results can also be influenced by moderate and fine resolution biogeophysical and social data, we analyzed these relationships using high resolution land cover classifications (1 m) as well as census tracts and census block groups. We hypothesized higher spatial resolution data would indicate stronger relationships among tree cover, income, and race/ethnicity due to increased accuracy in describing the heterogeneity of urban systems. Many studies have examined the relationships associated with UTC and demographics, using ordinary least squares regression (OLS), but because these variables are not randomly distributed across the landscape, we hypothesized that spatial autoregression (SAR) would provide more robust results.

## Materials and Methods

### City Site Descriptions

The seven case study cities vary in social (Table 1) and biogeophysical (Table 2) characteristics. Although high spatial resolution remotely sensed imagery is now freely available nationwide through the National Agriculture Imagery Program (NAIP), land cover classifications for cities using this imagery are still rare because they are expensive and time consuming to complete. The seven cities in our study were not randomly selected but based on the pooling of data from the NCEAS working group. Even though the cities are not stratified by social or biogeophysical characteristics, there are important differences among cities. Average annual temperatures fall within a band of 12.6 to 19°C (Table 2), and Los Angeles and Sacramento experience higher mean temperatures and receive less rainfall compared to the other cities. The arid cities of Los Angeles and Sacramento also have longer growing seasons and were not forested historically. In these regions, trees grew in and around riparian corridors along with oak forests in the surrounding foothills. For these California cities, water is a more important limiting factor to plant growth than the length of the growing season. In addition, with a predicted drying climate, Sacramento and Los Angeles are expected to experience declines in water supply. Although the East Coast cities (Baltimore, Raleigh, Philadelphia, New York City and Washington, D.C.) have longer winters and more freeze days annually, growing season increases slightly at the more southern locations, permitting higher growth rates and faster regrowth after removal of UTC. Existing UTC cover varies considerably across the case study cities, with the highest mean tree canopy percentage by CBG in Raleigh, NC (55%) and the lowest in Philadelphia, PA (13%) (Table 2).

**Table 1:** Site Descriptions—Social variables for the seven study cities.

	Population (2000)	Percent Asian (2000)	Percent Black (2000)	Percent White (2000)	Percent Hispanic (2000)	Median Income (1999)	No HS Diploma (%)	BA Degree or Higher (%)
Baltimore, MD	651,154	1.5	64.3	31.6	1.7	30,078	31.6	19.1
Los Angeles, CA	3,694,820	10.0	11.2	46.9	46.5	36,687	33.4	25.5
New York, NY	8,008,278	9.8	26.6	44.7	27.0	38,293	27.7	27.4
Philadelphia, PA	1,517,550	4.5	43.2	45.0	8.5	30,746	28.8	17.8
Raleigh, NC	276,093	3.4	27.8	63.3	7.0	46,612	11.5	44.8
Sacramento, CA	407,018	16.6	15.5	48.3	21.6	37,049	22.7	23.9
Washington, D.C.	572,059	2.7	60.0	30.8	7.9	40,127	22.2	39.1

Please note that not all race categories are included and that respondents can select more than one race for the 2000 Census. Race and Hispanic origin are considered separate. Median income refers to household income.

**Table 2:** Site Descriptions—Biogeophysical variables for the seven study cities.

	Mean Tree Canopy % by CBG	Mean Annual Precip	Mean Annual Temp (°C)	Cooling Degree Days	Heating Degree Days	Median Spring Freeze Day (-2.2°C)	Median Fall Freeze Day (-2.2°C)	Growing Season Days*
Baltimore, MD	22.34	41.94	12.6	4720	1147	3/30	11/10	226
Los Angeles, CA	17.61	15.14	19	1506	928	0/00	0/00	365
New York, NY	16.35	49.69	12.6	4754	1151	3/25	11/28	249
Philadelphia, PA	12.65	42.05	12.9	4759	1235	3/26	11/19	239
Raleigh, NC	54.64	46.49	15.3	3431	1456	3/15	11/22	253
Sacramento, CA	23.66	17.93	16.2	2666	1248	1/7	12/24	352
Washington, D.C.	27.52	41.94	12.6	4720	1147	3/30	11/10	226

Population and demographics from American FactFinder (factfinder.census.gov). Climate data from NOAA 1980–2010 Climate Normals: http://cdo.ncdc.noaa.gov/cgi-bin/climatenormals/climatenormals.pl http://www.ncdc.noaa.gov/oa/climate/normals/usnormals.html

*Calculated as the number of days in between the median freeze days in fall and spring for each location.

The cities represent a range of sizes, from New York City and Los Angeles (population 8,008,278 and 3,694,820 in 2000) to much smaller cities such as Raleigh, North Carolina (population of 276,093 in 2000) (Table 1). The racial and ethnic makeup of the cities varies (Table 1). Over 88% of the population in Baltimore, Philadelphia, Raleigh, and Washington D.C. self-identify as either Black or White, and not Hispanic. In contrast, Los Angeles, Sacramento and New York City are more racially and ethnically diverse. Differences in median household income range from $30,078 in Baltimore to $46,612 in Raleigh. Populations vary in educational attainment (Table 1). For example, 33.4% of residents in the city of Los Angeles do not have a high school diploma, while only 11.5% do not in Raleigh. Raleigh has the highest percentage (44.8%) of residents with a Bachelor’s degree or higher compared to Philadelphia, which has the lowest percentage of residents with a Bachelor’s degree or higher (17.8%).

### Social Data

GIS data layers of census block groups (CBG) for the seven cities were derived from the 2000 US Census TIGER (Topographically Integrated Geographic Encoding and Referencing System) dataset. Only census block groups that were completely within the boundary of the cities were used in this study (census boundaries do not always align with municipal boundaries). The same boundary data layers were used as the common boundary for all geospatial operations. The number of block groups included in the analyses for each city are listed in Table 3. The social variables used in this study were calculated at the CBG level using data from the 2000 US Census. Social variables include indicators of race, ethnicity, income, and educational attainment. For race variables we used percent White, Black, and Asian and, for ethnicity, percent Hispanic. For income, we used median household income. For educational attainment (age 25+) we used percent with no high school diploma and percent with a bachelor’s degree or higher. Percent of houses occupied by renters, median housing age, the percent of the CBG classified as residential, and population density were also included in the analyses. Because our study focuses on environmental justice we highlight results for race, ethnicity, and income; the remaining variables act as controls in our regression analysis and are typical in empirical environmental justice research. In addition, the variables may further explain UTC cover density and distribution.

**Table 3:** Spearman’s Correlation Results.

	Baltimore		Los Angeles		New York		Philadelphia		Raleigh		Sacramento		Washington DC	
	CBG	Tract	CBG	Tract	CBG	Tract	CBG	Tract	CBG	Tract	CBG	Tract	CBG	Tract
Percent Asian	-0.01	-0.09	0.21**	0.17**	0.03**	-0.02	0.03	0.05	0.06	0.09	-0.21**	-0.35**	0.11*	0.06
Percent Black	-0.4	0.04	-0.32**	-0.33**	0.02	0.04	0.09**	0.08	-0.18*	-0.35**	-0.39**	-0.57**	-0.19**	-0.02
Percent Hispanic	-0.00	-0.09	-0.42**	-0.48**	-0.26**	-0.25**	-0.12**	-0.16**	-0.14	-0.17	-0.23**	-0.27*	-0.06	-0.08
Income	0.36**	0.38**	0.65**	0.67**	0.28**	0.23**	0.31**	0.45**	0.35**	0.38**	0.36**	0.31**	0.46**	0.32**
n	710	200	2449	839	5732	2216	1816	381	123	60	289	85	433	188

Note:

** p <. 01.

* p <. 05.

### Biogeophysical Data—High Resolution Tree Canopy

The percent of tree canopy cover for each CBG was calculated based on tree canopy data derived from high spatial resolution imagery. The imagery used included 0.6 m resolution pan-sharpened QuickBird satellite imagery, 1 m resolution NAIP (National Agriculture Imagery Program) near-infrared aerial imagery, and 0.15 m resolution natural color aerial imagery. LiDAR (light detection and ranging) data with 0.5 m or 1 m resolution were also used to aid in classification. Object-based classification approaches were used for tree canopy classification for all cities, except Los Angeles, where a pixel-based approach was used. Overall accuracy for the New York City classification was 96%, Sacramento was 92%, Philadelphia was 95%, and Baltimore was 94%. The accuracy of the Los Angeles tree cover classification was assessed using a stratified random sample of 100 parcels across the city, with an overall classification accuracy of 88.6% based on pixel-by-pixel comparison [48]. Although overall accuracy was not calculated for the remaining cities, similar data and procedures were used and we assume high accuracy for those classifications as well.

### Statistical Analyses

Bivariate and multivariate techniques were used in the analyses. Spearman’s correlation, ordinary least squares (OLS) regressions, and spatial autoregressive (SAR) techniques were used to investigate the relationship between key environmental justice variables (race, ethnicity, and income) and UTC cover. The CBG served as the unit of analysis. The percent of UTC cover was used as the response variable in subsequent statistical analyses.

Bivariate analysis and correlations between major variables has been a primary analytical tool in EJ studies. It is used to establish a baseline incidence of disparities in the distribution of environmental bads (or goods) and particular race, income and demographic characteristics. In our analysis, we use the Spearman correlation measure as a simple indicator of association between two variables (e.g. high values in one matching high values in another). We employ this type of analysis in our paper in order to formulate a baseline “picture” that is comparable across all cities in our analysis.

We next employ OLS in order to correct some of the obvious disadvantages that emerge from bivariate analysis. Regression allows us to examine the effect of a change in one variable on the value of another variable, controlling for other factors in the system under study. Empirically, the correlation coefficient between two variables can be different from a multiple regression coefficient when one of the variables is the dependent variable and the other, the independent variable. There is a theoretical possibility that a correlation coefficient between two variables is the same with the one you would get in regression analysis, but that happens only in the case of simple regression (one dependent and one independent variable) and only if the standard deviations of the two variables coincide. In multiple regression, the researcher captures the effects of other covariates (beyond the two that are included in the bivariate analysis) with the result of different correlation and regression coefficients. Thus, results from bivariate and multivariate (regression) analysis are expected to be different and not directly comparable. Moving away from simple co-variation, the latter is a methodology that tries to get closer to causation (but still is not causation).

The OLS method assumes that the error terms are independent. From a spatially-explicit perspective, this is an unrealistic assumption since according to Tobler’s Law [49], “everything is related to everything else, but near things are more related than distant things.” Many studies employing spatial data are typically hindered by the problem of spatial autocorrelation, which biases coefficient estimates of the variables employed in OLS regressions—typically described as providing best linear unbiased estimators [45].

Identification of spatial autocorrelation patterns requires the use of spatial autoregression modeling and the comparison of the results from the two regression approaches. Anselin [45] classifies spatial econometric models in two broad categories: models of spatial dependence (capturing auto-correlative effects of distance in spatial processes) and spatial heterogeneity (capturing the lack of stability or relationships across space). Spatial autoregression methods bring the spatial interrelationship structure of the units of analysis into the standard multiple regression in order to correct for problems of spatial autocorrelation. They quantify the spatial relationship of a (dependent) variable by a n×n matrix of spatial weights with each element of the matrix representing the strength of the interaction of two locations based on a proximity measure such as contiguity or distance. Spatial regression models have become increasingly popular in a variety of fields such as geography, economics, and political science [50], demography [51], ecology [52], and other fields that rely on spatially explicit data.

There are two spatial autoregressive techniques that have foundations in theoretical and practical considerations: the spatial lag (SLAG) model and spatial error (SEM) model [45]. The SLAG assumes that the imposed spatial structure affects the dependent variable, introducing a spatially lagged dependent variable. Typical theoretical explanations of the importance of the SLAG include issues of externalities and spill-over effects. In our study, socioeconomic conditions, local climatology, and ecological processes could account for such effects. A first order mixed regressive-spatial autoregressive SLAG model takes the matrix algebra model form of:

y=ρWy+Xβ+ε (1)

where *Wy* is a *n×1* vector of the spatially lagged response variable, *ρ* is the spatial autoregressive coefficient, *X* is a *n×k* vector of explanatory variables, *β* is the *k×1* vector of regression coefficients and *ε* is a *n×1* vector of independently and identically distributed (iid) errors.

The SEM model incorporates the spatial effects into the error terms capturing the spatial structure of unobserved variation e.g. from missing variables that are spatially autocorrelated. This model is essentially capturing a spatial form of the problem of heteroscedasticity. The SEM model takes the mixed regressive—autoregressive form of

$$y=X\beta +{\left(I-\lambda W\right)}^{-1}\varepsilon$$ (2)

where λ is the spatial autoregressive coefficient, W is the n×n spatial weight matrix, I is the n×n identity matrix and ε is a n×1 vector of iid errors. This paper reports the first type of spatial modelling (spatial dependence) using spatial autoregression methods.

We use a queen contiguity-based spatial weight matrix *W* (in the order of one) for all cities in our study. This approach in creating spatial weight matrices imposes a spatial structure in the units of observation that takes into account the topology of shared borders or vertices of the irregular polygon data. All regression models and spatial statistics used for measuring spatial autocorrelation are estimated using maximum likelihood estimation and are run on GeoDa 0.9.5-i software platform [53].

The spatial models are compared through the Akaike Information Criterion (AIC) and log-likelihood statistic [45]. A higher value of log-likelihood and a lower value of AIC point to a model with a better fit. Only the model with the best fit (using spatial lag or spatial error) is reported in the results. The model with the best fit is indicated in table 4.

**Table 4:** Ordinary Least Square (OLS) and Spatial Autoregressive (SAR) Results.

	Baltimore		Los Angeles		New York		Philadelphia		Raleigh		Sacramento		Washington DC	
	OLS	SAR (SLAG)	OLS	SAR (SEM)	OLS	SAR (SEM)	OLS	SAR (SLAG)	OLS	SAR (SEM)	OLS	SAR (SEM)	OLS	SAR (SLAG)
Percent Asian	-0.10	-0.17	-0.03*	-0.03*	-0.05**	-0.08**	-0.00	-0.01	-0.03	-0.04	-0.12**	0.01	0.10	0.08
Percent Black	0.07**	0.02*	-0.10**	-0.01	0.01*	0.02*	0.05**	0.03**	0.13*	0.11	-0.11*	-0.02	0.04	0.05
Percent Hispanic	-0.10	0.03	0.03*	-0.00	-0.00	-0.04**	0.04**	0.03*	0.12	0.11	0.03	0.03	0.24**	0.09
Income (in thousands)	0.09*	0.05	0.07**	0.07	-0.05**	-0.04**	0.17**	0.09**	-0.18*	-0.17*	0.03	0.10**	0.20**	0.10**
R squared	0.56	0.75	0.54	0.82	0.18	0.42	0.40	0.60	0.55	0.56	0.56	0.79	0.46	0.70
n	710	710	2449	2449	5732	5732	1816	1816	123	123	289	289	433	433

Note: Results are shown for Census Block Group.

** p <. 01.

* p <. 05.

## Results

### Bivariate Analysis

**Fig 1:**
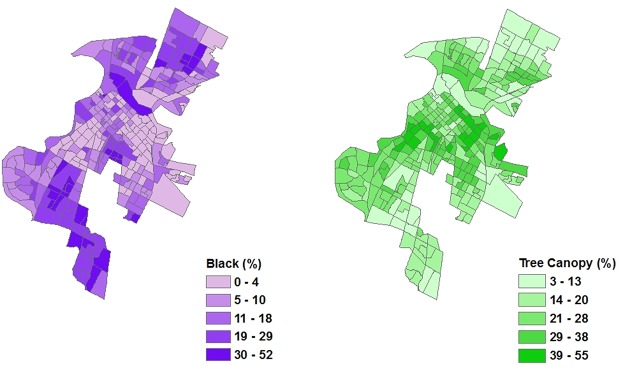
A spatially-explicit map depicting the percent of the population that self-identifies as black (left panel) and the percent of UTC cover for Sacramento City, CA (right panel).

**Fig 2:**
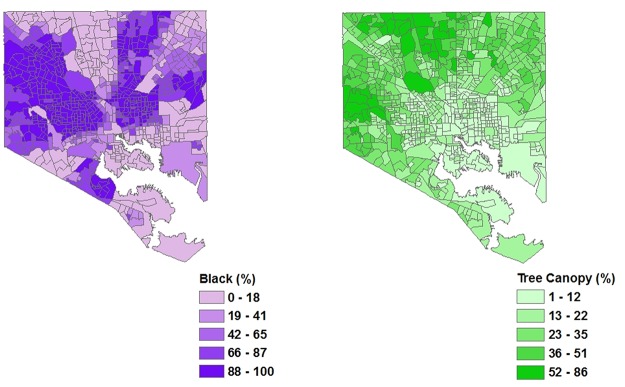
A spatially-explicit map depicting the percent of the population that self-identifies as black (left panel) and the percent of UTC cover for Baltimore, MD (right panel).

In some cities, the bivariate analyses reveal negative and significant relationships among race, ethnicity, and UTC cover. However, the signs of the estimated coefficients are not consistent across all cities. Specifically, the relationship between UTC cover and percent Asian is positive and significant for Los Angeles and negative and significant for Sacramento (Table 3). The relationship between UTC cover and percent Black is negative and significant for Los Angeles, Raleigh, Sacramento, and Washington D.C. (Table 3, Figs. 1 and 2). The relationship between UTC cover and percent Hispanic is negative and significant for Los Angeles, New York City, Philadelphia, and Sacramento (Table 3). Consistent across all cities is a positive and significant relationship between UTC cover and median household income (Table 3).

### Multivariate Analyses

In general, the multivariate regressions reveal that with the addition of other control variables the relationships between race or ethnicity and UTC are not as strong likely due to collinearity among the variables included in the regression. Negative and statistically significant associations between percent Asian and UTC cover are observed in Los Angeles and New York City although the regression coefficients are very small (Table 4). In Baltimore, Philadelphia, and New York City, there is a significant and positive association between percent Black and UTC cover but regression coefficients are very small (Table 4). Relationships between percent Black and UTC are significant and weakly positive in Raleigh and negative in Los Angeles but only using OLS models; they are not significant using SAR models. Finally, there is a significant and positive association between percent Hispanic and UTC cover in Los Angeles (OLS), Philadelphia (OLS & SAR), and Washington D.C. (OLS) although the regression coefficients are very small (Table 4).

Income, which was positively correlated with UTC and highly significant across all cities in the bivariate analyses, shows positive and statistically significant in most cities for the multivariate regressions but the magnitude of the coefficients is not large (Table 4). We estimate that an increase of median household income by $1,000 (in 2000 dollars) brings about a range of 0.05–0.20 point increase in the percent of UTC for the majority of our cities. Philadelphia and Washington D.C. present the highest positive income coefficients. New York and Raleigh are the only two cities with a negative effect of income; the magnitude of that effect is very close to zero for New York—even if statistically significant—while it is more substantial in the case of Raleigh.

We also examine the effects of income through the use of SAR models. The SAR models that account for the spatial structure of the data consistently provide more robust results compared to the OLS models, as evidenced by higher r-square values across all cities (Table 4). Overall, while the SAR models do not substantially alter our findings about the signs of income coefficients, differences occur in terms of magnitude and statistical significance. In the case of Los Angeles, the statistical significance of the estimated positive income coefficient is reduced. The SAR models also reduce the size of the positive income coefficient for Philadelphia and Washington, D.C. In the case of Raleigh, the difference between the estimated income coefficients for OLS models and the SAR models are minimal. Our findings suggest collectively that in addition to our variables exhibiting collinearity they are also spatially clustered.

## Discussion

Correlations between the distribution of benefits and socio-economic variables can vary across cities. Baltimore is not the same as Philadelphia, but the cities may share policies and regulations that have the potential to create environmental injustice. However, factors such as climate, demographics, and city size may filter the outcome that shared processes have on the distribution of environmental amenities and burdens. Demographics or, more specifically, racial and ethnic diversity differs among the represented cities and may represent an alternative explanation to climate. Along with an arid climate, Sacramento and Los Angeles have greater racial and ethnic diversity compared to the other cities with a larger portion of the population self-identifying as Asian or Hispanic. The only city in the Northeast with comparable racial and ethnic diversity is New York City, which demonstrates negative correlations between UTC cover and ethnicity, but not race. We have approached our analyses using various statistical methods, each one revealing something new about our data. The most significant finding from each method is listed in Table 5.

**Table 5:** Statistical Methods Used.

Question	Statistical Method	Finding	Implication
Is UTC cover distributed equally in the cities examined?	Spearman’s Correlation	Positive correlation with income across all cities. Strongest correlations among UTC and race occur in arid cities.	Regardless of what drives the pattern, the pattern exists—UTC cover is not equally distributed in regards to income, and in some cases, race.
What other variables drive the distribution of UTC cover?	Ordinary Least Squares Regression	There are no strong consistent drivers across all cities.	This is likely due to collinearity among the variables included in the regression providing less explanatory power.
Do the data have significant spatial structure?	Spatial Autoregressive Model	SAR models result in a better fit, evidenced by higher r-square values.	This suggests that in addition to our variables exhibiting collinearity, they are also spatially clustered. Accounting for the spatial structure improves fit.

Cities are patchy landscapes, and this unevenness can lead to inequitable distribution of environmental benefits and burdens [54,55]. We expected all of the seven cities to show that neighborhoods with lower-income, less education, and high percentages of people of color would have low UTC cover. Overall, our results confirm that UTC cover is related to social characteristics of neighborhoods but not consistently across cities that vary in climate, size, and racial and ethnic composition. We also found that the ability to detect patterns in the data is affected by statistical method (OLS vs. SAR). Accounting for spatial autocorrelation produces more robust models and in certain cases corrects the aspatial specification bias of regular OLS estimation (Table 4).

The most striking pattern in the data comes from the bivariate analyses which reveal a strong positive relationship between UTC cover and income across all cities. Our multivariate regression analysis, controlling for other factors, reveals a positive relationship between UTC cover and income for most cities in our study (with the exception of New York and Raleigh). As our methodology section explains, it is theoretically possible to have two variables with positive correlations through bivariate analysis that become negatively related in a multiple regression context; controlling for other variables adjusts for the potential bias in the coefficient of the income variable, with the resulting sign reversal in the case of two cities in our study. The relationship between UTC cover and income may be the result of a feedback loop where high amounts of UTC cover increase property values and further attract households with high incomes. This positive feedback loop may support the continued maintenance of UTC cover in neighborhoods with high-income households and high levels of homeownership. Similarly, areas with low UTC cover have low property values, and residents may have less access to resources or incentive to increase property values because they are renters or on fixed incomes. Residents in low income neighborhoods might reasonably resist increases in UTC cover to avoid gentrification and rising rents [56]. The cost of tree maintenance, such as leaf clean-up, watering, and pruning, is another disincentive that can be particularly acute in low-income neighborhoods [33].

Income is so strongly associated with UTC cover that it must be controlled for in the analyses in order to detect additional drivers. Race/ethnicity was not consistently a significant factor in the bivariate analyses (Table 3) and few factors in addition to income were significant in the multivariate analyses (Table 4). However, in the bivariate analyses of our California cities, we did see evidence of a link between race/ethnicity and UTC (Table 3). For Sacramento and Los Angeles, percent black and percent Hispanic are strongly and negatively correlated with UTC cover, but the relationships are weak or insignificant for the other cities. A distinguishing characteristic of the California cities is their arid climate. Trees planted in more arid climates require irrigation in order to survive, while in temperate regions of the United States, trees can grow without water subsidies on unmanaged lands. Given the high water resource requirements in arid cities, the dominant role of income in driving UTC cover likely creates a greater potential for environmental injustice in cities receiving little precipitation. Natural growth and regeneration of trees in cities with greater precipitation could dampen any effects of different levels of resources allocated towards growing and maintaining UTC cover.

In arid climates, trees place a direct demand on limited resources, such as water, and an indirect demand on additional resources, such as energy used to transport that water, making trees more costly from an environmental, social, and economic perspective. In arid climates, treeless areas may represent less investment in UTC cover and/or management, while in temperate areas, treeless areas may not be a reliable indicator of low investment. In contrast, treeless areas may represent areas of greater economic and/or community investment, such as the costs of maintaining grassy areas. Nevertheless, the results of this study cannot be interpreted without consideration of some important caveats, which we outline below.

### Other variables may be better predictors of tree canopy cover

This study was framed by environmental justice, which focuses on race and ethnicity as well as other social status characteristics (e.g., income, education, homeownership) as the variables of concern. While the models included population density and housing density as proxies for building density, other variables such as urban morphology [57], impervious surface, land rents, past tree canopy cover, water budgets, tree planting policies, and dominant tree species might generate more robust models.

### Examining patterns between race and amenities does not capture intent

Identifying patterns of inequity are important and bring attention to the inequitable distribution of environmental goods or bads but it is equally important to understand the processes driving the inequitable distribution. Consideration of both distributional and procedural equity can help explain why the expected patterns predicted by environmental justice theory are not always present [15,58,59]. For instance, the high amount of green vegetation in predominantly African American neighborhoods in Baltimore may be reflective of the increased number of vacant lots in those neighborhoods, which resulted in part from decades of deliberate disinvestment and discrimination [6,60].

### Vegetation structure and social structure may be mismatched

Our study is a comparison of current vegetation structure and how it relates to current social structure. However, trees are long-lived organisms that can take a very long time to establish and grow. In contrast, the social structure of cities can change more rapidly. Recent studies have highlighted this phenomenon describing the current landscapes we observe as legacies of past consumption patterns [11]. Specifically Boone et al. [11] found that vegetation operating at short time scales (i.e. lawns) reflect current lifestyle characteristics, while vegetation that takes longer to establish (i.e. trees) are reflections of the characteristics of past residents. Research that does not include a historical analysis is still valuable as it represents current patterns and potential inequities associated with environmental amenities, but it is also important to keep in mind that present day patterns could be the result of inherited landscapes. This may be especially true for systems where trees are not part of the native landscape and are typically planted at the time of development. A more complete picture of the correlations between tree canopy cover and social characteristics could be obtained from analyzing multiple time periods that correspond to the life expectancy of dominant species of trees in the study area. However, incorporating the importance of legacies and inherited landscapes into studies such as these requires multiple and compatible datasets.

### Tree cover canopy is treated as homogeneous across the unit of analysis

In reality, tree canopy cover may be clustered with some residents in census block groups experiencing greater coverage and others experiencing less. In addition, our analysis does not distinguish between tree canopy cover on public versus private lands or residential versus non-residential lands. This may be important as management regimes likely differ on public versus private and residential versus non-residential lands [43]. This distinction may also speak to the total plantable surfaces that are available for increasing UTC cover. In addition, by not distinguishing between residential and non-residential we may be inflating or decreasing tree canopy cover. For a CBG that contains a small residential area and a large park, the sum of all the trees within the tract will be correlated with the social characteristics that correspond to only the residential area of the CBG.

### Trees are not always an environmental amenity

While trees are generally accepted as an environmental amenity, evidenced by the many public and private investments supporting increased tree canopy cover; trees are sometimes considered a disamenity, especially in places of disinvestment [9]. Trees may place a direct demand on limited resources, such as water, and an indirect demand on additional resources, such as energy used to transport water. In addition, there is the *perception* that increased vegetation facilitates crime [36,61] although recent research in Baltimore has shown a decrease in crime with increasing UTC cover [62]. Residents may also incur maintenance costs [36]. Trees can also be a source of allergens and pollution precursors (VOCs). While some of the benefits attributed to increased tree canopy cover are well supported in the scientific literature, others, such as the removal of atmospheric pollutants, are more tenuous [34]. For many of the purported ecosystem services attributed to increased tree cover the data simply do not exist in order to adequately evaluate and quantify the service provided [34]. More research is needed in order to accurately evaluate the ecosystem services provided by increased tree canopy cover and evaluate the tradeoffs.

## Conclusion

Money may not grow on trees, but this study suggests that in a way, trees grow on money. Our findings show that high-income neighborhoods in our selected cities are more likely than low-income neighborhoods to have high tree canopy cover. We did not find, however, the expected pattern of low tree canopy cover in neighborhoods with a high percentage of racial and ethnic minorities for all cities; only in the California cities of Sacramento and Los Angeles did we see such a result. Since these cities depend on irrigation to maintain tree canopy cover, this distinguishing factor of aridity may amplify socioeconomic differences in neighborhood tree cover more than in the humid temperate cities we analyzed. In-depth analyses of the costs and benefits of maintaining tree cover in arid and humid environments along with qualitative analyses of residential land management practices could provide some explanation for these differences.

Increasing UTC has become a widespread goal, often incorporated into municipal sustainability plans. It has been proposed as a way to mitigate impacts from human-dominated systems on the immediate (e.g. shade and cooling) and global (e.g. carbon capture) environment. Sacramento Tree Foundation has pledged to plant five million trees by the year 2025, an effort that would double the region’s tree canopy cover. Philadelphia has established a goal of increasing tree canopy cover to 30% by the year 2025 (www.phila.gov/green/trees). New York City, Baltimore, and Los Angeles have also announced extensive tree planting initiatives (www.milliontreesnyc.org, www.baltimorecity.gov, www.milliontreesla.org). In addition to regional efforts, there are national and global efforts to bring more awareness to the benefits of UTC cover (Urban Environmental Accord 2005, www.sfenvironment.org/downloads/library/accords.pdf; http://www.plant-for-the-planet-billiontreecampaign.org/Partners/VariousPartners/TreePlanting.aspx). One of the implications of embedding tree canopy goals in sustainability plans is that environmental justice is frequently included as an objective of the plans, and sometimes explicitly linked to UTC. For example, Philadelphia’s 2009 GreenWorks Plan includes goals of increasing tree canopy cover *in all neighborhoods* highlighting the desire for the equitable distribution of UTC cover (www.phila.gov/green/greenworks/2009-greenworks-report.html).

If the equity dimensions of sustainability are put into practice, UTC goals can help to redress environmental injustices. However, it is important to note that tree planting schemes are not a panacea for environmental justice. Often overlooked is that the presence of trees can generate disservices, and in some cases costs may exceed local benefits or local desire and capacity to care for trees and other green infrastructure [33,34,63]. Benefits from tree canopy cover must therefore be assessed in spatially explicit ways, since in some circumstances tree cover will provide strong positive services while in others tree cover may provide negligible benefits or disservices.

The association between income and tree canopy cover has important implications for urban sustainability plans, many of which include increased UTC cover as a goal. If UTC and income are positively reinforced (through property values, investments, or other mechanisms) any public or private interventions to increase tree canopies might first consider the needs of low income communities, provided that the economic and ecological benefits do not outweigh the costs and that the initiatives are supported by local residents. In addition to considering distributional equity, urban sustainability goals need to be supported by stronger science on perceived ecosystem services provided by UTC cover so that justice can be considered in terms of the distribution of ecosystem services, not just the equitable distribution of trees.

## Supporting Information

S1 Table Full Model Results. The full model results of the OLS and spatial regressions at the CBG level omitting the intercept. Median household income is reported in dollars.
(DOCX)
