# Supplementary material for: Trees Grow on Money: Urban Tree Canopy Cover and Environmental Justice
Source: PLoS One. 2015 Apr 1;10(4):e0122051. doi: 10.1371/journal.pone.0122051 (PMC4382324; doi:10.1371/journal.pone.0122051)
Supplement: S1 Table — The full model results of the OLS and spatial regressions at the CBG level omitting the intercept. Median household income is reported in dollars. (DOCX) [file pone.0122051.s001.docx]

**S1 Table. Full Model Results.** The full model results of the OLS and spatial regressions at the CBG level omitting the intercept. Median household income is reported in dollars.

**Baltimore, MD OLS**

R square: 0.56

Number of Observations: 710

| Variable | Coefficient | P value |
| --- | --- | --- |
| Percent Asian | -0.09865593 | 0.4708787 |
| Percent Black | 0.06635435 | 0.0000023 |
| Percent Hispanic | -0.09514766 | 0.4156902 |
| Percent Bachelor’s or Higher | 0.2316971 | 0.0000000 |
| Percent No High School | -0.05814618 | 0.1346190 |
| Median Household Income | 8.554848e-005 | 0.0267933 |
| Percent Renter Occupied | 0.1323241 | 0.0000000 |
| Housing Density | -0.003158398 | 0.0000000 |
| Median Housing Age | -0.01110426 | 0.0000043 |
| Percent Residential | 0.319631 | 0.0000000 |
| Population Density | -0.0007175005 | 0.0000013 |

**Baltimore, MD SAR (SLAG)**

R square: 0.75

Number of Observations: 710

| Variable | Coefficient | P value |
| --- | --- | --- |
| Percent Asian | -0.1694023 | 0.0996490 |
| Percent Black | 0.02301605 | 0.0285573 |
| Percent Hispanic | 0.02626179 | 0.7653314 |
| Percent Bachelor’s or Higher | 0.1322238 | 0.0000062 |
| Percent No High School | 0.006274743 | 0.8312878 |
| Median Household Income | 5.453843e-005 | 0.0603328 |
| Percent Renter Occupied | 0.07997306 | 0.0000016 |
| Housing Density | -0.00126021 | 0.0000075 |
| Median Housing Age | -0.008362998 | 0.0000036 |
| Percent Residential | 0.1221853 | 0.0000000 |
| Population Density | -0.0004504699 | 0.0000492 |

**Los Angeles, CA OLS**

R square: 0.54

Number of Observations: 2449

| Variable | Coefficient | P value |
| --- | --- | --- |
| Percent Asian | -0.03447574 | 0.0256903 |
| Percent Black | -0.100818 | 0.0000000 |
| Percent Hispanic | 0.03121855 | 0.0416715 |
| Percent Bachelor’s or Higher | 0.1819017 | 0.0000000 |
| Percent No High School | -0.05586807 | 0.0052837 |
| Median Household Income | 7.002071e-005 | 0.0000000 |
| Percent Renter Occupied | -0.03134991 | 0.0023170 |
| Housing Density | -0.001692299 | 0.0000000 |
| Median Housing Age | -0.001255932 | 0.2331398 |
| Percent Residential | 1.425553 | 0.0000000 |
| Population Density | 0.0001703862 | 0.1312023 |

**Los Angeles, CA SAR (SEM)**

R square: 0.82

Number of Observations: 2449

| Variable | Coefficient | P value |
| --- | --- | --- |
| Percent Asian | -0.02908873 | 0.0429848 |
| Percent Black | -0.009505632 | 0.5179749 |
| Percent Hispanic | -0.00409466 | 0.7372137 |
| Percent Bachelor’s or Higher | 0.1173555 | 0.0000000 |
| Percent No High School | -0.02333432 | 0.1086863 |
| Median Household Income | 7.420392e-006 | 0.3870335 |
| Percent Renter Occupied | -0.03290984 | 0.0000130 |
| Housing Density | -0.001308047 | 0.0000000 |
| Median Housing Age | -0.0005290653 | 0.4598202 |
| Percent Residential | 1.267915 | 0.0000000 |
| Population Density | 7.981155e-005 | 0.3246201 |

**New York City, NY OLS**

R square: 0.18

Number of Observations: 5732

| Variable | Coefficient | P value |
| --- | --- | --- |
| Percent Asian | -0.04702724 | 0.0000032 |
| Percent Black | 0.01095483 | 0.0208121 |
| Percent Hispanic | -0.002188744 | 0.7637434 |
| Percent Bachelor’s or Higher | 0.1183658 | 0.0000000 |
| Percent No High School | -0.0541594 | 0.0000183 |
| Median Household Income | -4.983553e-005 | 0.0000001 |
| Percent Renter Occupied | -0.04463426 | 0.0000000 |
| Housing Density | -0.0002112892 | 0.0000004 |
| Median Housing Age | -0.001323661 | 0.0276709 |
| Percent Residential | 0.1535623 | 0.0000000 |
| Population Density | -8.834976e-006 | 0.6585055 |

**New York City, NY SAR (SEM)**

R square: 0.42

Number of Observations: 5732

| Variable | Coefficient | P value |
| --- | --- | --- |
| Percent Asian | -0.0779728 | 0.0000000 |
| Percent Black | 0.01797417 | 0.0130568 |
| Percent Hispanic | -0.03748614 | 0.0000308 |
| Percent Bachelor’s or Higher | 0.078313 | 0.0000000 |
| Percent No High School | -0.004395142 | 0.6975087 |
| Median Household Income | -3.882175e-005 | 0.0000108 |
| Percent Renter Occupied | -0.009087931 | 0.1971653 |
| Housing Density | -8.598879e-005 | 0.0541989 |
| Median Housing Age | -0.001792347 | 0.0010110 |
| Percent Residential | 0.131136 | 0.0000000 |
| Population Density | -8.37889e-005 | 0.0000466 |

**Philadelphia, PA OLS**

R square: 0.40

Number of Observations: 1816

| Variable | Coefficient | P value |
| --- | --- | --- |
| Percent Asian | -0.002619466 | 0.9130164 |
| Percent Black | 0.05285299 | 0.0000000 |
| Percent Hispanic | 0.0388327 | 0.0045986 |
| Percent Bachelor’s or Higher | 0.1391117 | 0.0000000 |
| Percent No High School | -0.04905669 | 0.0049420 |
| Median Household Income | 0.0001675523 | 0.0000000 |
| Percent Renter Occupied | 0.07002264 | 0.0000000 |
| Housing Density | -0.0007096402 | 0.0001674 |
| Median Housing Age | -0.003258964 | 0.0001590 |
| Percent Residential | 0.1180562 | 0.0000000 |
| Population Density | -0.0004932415 | 0.0000006 |

**Philadelphia, PA SAR (SLAG)**

R square: 0.60

Number of Observations: 1816

| Variable | Coefficient | P value |
| --- | --- | --- |
| Percent Asian | -0.01338241 | 0.4927785 |
| Percent Black | 0.02670228 | 0.0000000 |
| Percent Hispanic | 0.02816893 | 0.0115499 |
| Percent Bachelor’s or Higher | 0.0518488 | 0.0001805 |
| Percent No High School | -0.03852974 | 0.0067634 |
| Median Household Income | 9.323095e-005 | 0.0000000 |
| Percent Renter Occupied | 0.02667037 | 0.0029457 |
| Housing Density | -0.0002589358 | 0.0915474 |
| Median Housing Age | -0.0007899644 | 0.2621500 |
| Percent Residential | 0.009511328 | 0.4237571 |
| Population Density | -0.0002074168 | 0.0104884 |

**Raleigh, NC OLS**

R square: 0.55

Number of Observations: 123

| Variable | Coefficient | P value |
| --- | --- | --- |
| Percent Asian | -0.02515213 | 0.9383915 |
| Percent Black | 0.1268416 | 0.0304209 |
| Percent Hispanic | 0.1223582 | 0.3174488 |
| Percent Bachelor’s or Higher | 0.08951561 | 0.3628973 |
| Percent No High School | -0.1610129 | 0.2077138 |
| Median Household Income | -0.0001786685 | 0.0289689 |
| Percent Renter Occupied | -0.1539209 | 0.0093991 |
| Housing Density | -0.006663367 | 0.1160905 |
| Median Housing Age | 0.007863337 | 0.1747404 |
| Percent Residential | 0.4096791 | 0.0000000 |
| Population Density | 0.002256158 | 0.2529118 |

**Raleigh, NC SAR (SEM)**

R square: 0.56

Number of Observations: 123

| Variable | Coefficient | P value |
| --- | --- | --- |
| Percent Asian | -0.03859736 | 0.9013044 |
| Percent Black | 0.1051943 | 0.0732561 |
| Percent Hispanic | 0.1113269 | 0.3274092 |
| Percent Bachelor’s or Higher | 0.06047299 | 0.5284501 |
| Percent No High School | -0.1587924 | 0.2077453 |
| Median Household Income | -0.0001725719 | 0.0255022 |
| Percent Renter Occupied | -0.1539177 | 0.0054694 |
| Housing Density | -0.006580671 | 0.1053713 |
| Median Housing Age | 0.008441119 | 0.1302369 |
| Percent Residential | 0.4111514 | 0.0000000 |
| Population Density | 0.002274611 | 0.2181045 |

**Sacramento, CA OLS**

R square: 0.56

Number of Observations: 289

| Variable | Coefficient | P value |
| --- | --- | --- |
| Percent Asian | -0.1245937 | 0.00083 |
| Percent Black | -0.1092469 | 0.01126 |
| Percent Hispanic | 0.02616373 | 0.55740 |
| Percent Bachelor’s or Higher | 0.2993236 | 0.00000 |
| Percent No High School | 0.1203542 | 0.01446 |
| Median Household Income | 3.052681e-005 | 0.56198 |
| Percent Renter Occupied | 0.02355939 | 0.45280 |
| Housing Density | 0.003322599 | 0.00067 |
| Median Housing Age | -0.006061257 | 0.04170 |
| Percent Residential | 0.1970188 | 0.00000 |
| Population Density | -0.0005213956 | 0.07004 |

**Sacramento, CA SAR (SEM)**

R square: 0.79

Number of Observations: 289

| Variable | Coefficient | P value |
| --- | --- | --- |
| Percent Asian | 0.01273681 | 0.68077 |
| Percent Black | -0.02369551 | 0.54529 |
| Percent Hispanic | 0.03276404 | 0.31286 |
| Percent Bachelor’s or Higher | 0.1579269 | 0.00015 |
| Percent No High School | -0.03080654 | 0.40487 |
| Median Household Income | 0.0001039211 | 0.00296 |
| Percent Renter Occupied | 0.01373081 | 0.51988 |
| Housing Density | 0.002616896 | 0.00084 |
| Median Housing Age | -0.003613357 | 0.06247 |
| Percent Residential | 0.1828874 | 0.00000 |
| Population Density | -0.0002772249 | 0.14260 |

**Washington, DC OLS**

R square: 0.46

Number of Observations: 433

| Variable | Coefficient | P value |
| --- | --- | --- |
| Percent Asian | 0.1015783 | 0.6215174 |
| Percent Black | 0.03952639 | 0.4409423 |
| Percent Hispanic | 0.2388635 | 0.0097067 |
| Percent Bachelor’s or Higher | 0.07101428 | 0.3453675 |
| Percent No High School | -0.166635 | 0.0512760 |
| Median Household Income | 0.0002001232 | 0.0000005 |
| Percent Renter Occupied | 0.1154585 | 0.0009326 |
| Housing Density | -0.0007224304 | 0.3126912 |
| Median Housing Age | -0.006354714 | 0.1403559 |
| Percent Residential | 0.288331 | 0.0000000 |
| Population Density | -0.001309582 | 0.0022574 |

**Washington, DC SAR (SLAG)**

R square: 0.70

Number of Observations: 433

| Variable | Coefficient | P value |
| --- | --- | --- |
| Percent Asian | 0.07901413 | 0.6021071 |
| Percent Black | 0.0459065 | 0.2246929 |
| Percent Hispanic | 0.09276362 | 0.1711534 |
| Percent Bachelor’s or Higher | 0.0318401 | 0.5660820 |
| Percent No High School | -0.05936688 | 0.3451256 |
| Median Household Income | 9.604378e-005 | 0.0009649 |
| Percent Renter Occupied | 0.02772408 | 0.2782260 |
| Housing Density | 7.992388e-005 | 0.8797373 |
| Median Housing Age | -0.0036835 | 0.2454111 |
| Percent Residential | 0.03903467 | 0.2150817 |
| Population Density | -0.0005928335 | 0.0597203 |
